# Tobacco and Nervous System Development and Function—New Findings 2015–2020

**DOI:** 10.3390/brainsci11060797

**Published:** 2021-06-16

**Authors:** Wojciech Hajdusianek, Aleksandra Żórawik, Marta Waliszewska-Prosół, Rafał Poręba, Paweł Gać

**Affiliations:** 1 Department of Hygiene, Wroclaw Medical University, 50-345 Wroclaw, Poland; wojciech.hajdusianek@student.umed.wroc.pl (W.H.); aleksandra.zorawik@student.umed.wroc.pl (A.Ż.); 2 Department of Neurology, Wroclaw Medical University, 50-556 Wroclaw, Poland; marta.waliszewska@umed.wroc.pl; 3 Department of Internal Medicine, Occupational Diseases, Hypertension and Clinical Oncology, Wroclaw Medical University, 50-556 Wroclaw, Poland; rafal.poreba@umed.wroc.pl

**Keywords:** environmental tobacco smoke, nervous system, brain

## Abstract

Tobacco is a one of the most common addictive stimulants used by people around the world. The smoke generated during tobacco combustion is a toxic mixture of more than 5000 chemicals of which over 30 are known human carcinogens. While its negative effects on the human body are well understood, it remains a serious public health problem. One of the multiple effects of smoking is tobacco’s effect on the nervous system—its development and function. This review aims to summarize the progress made in research on the effects of tobacco on the nervous system both of the perinatal period and adults and both in animals and humans in 2015–2020. The 1245 results that corresponded to the keywords “tobacco, cigarette, nervous system, brain, morphology, function” were reviewed, of which 200 abstracts were considered significant. Most of those articles broadened the knowledge about the negative effects of smoking on the human nervous system. Tobacco has a significant negative impact on the development of nervous structures, neurotransmission and cognitive functions, and promotes the development of neurodegenerative diseases, insomnia and cerebrovascular diseases. The only exception is the protective effect of the dopaminergic system in Parkinson’s disease. In conclusion, in recent years much effort has been devoted to describing, revealing and uncovering new aspects of tobacco detrimental to human life. The nicotine contained in tobacco smoke affects the human body in a multidimensional way, including a serious impact on the broadly understood neurological health.

## 1. Introduction

According to a WHO global report on trends of tobacco use, by the year of 2025 the prevalence of tobacco use will be 30% in males and 8% in females [1]. While Tobacco has been a subject of research for quite a long time (the oldest indexed scientific paper with key word Tobacco was published in 1807 [2],) there are some fields of its relation to health that still need to be discovered. The ETS was subject to numerous reviews; some of them were focused on cognition [3], some paid more attention to molecular mechanisms [4], or even neural circuits and receptors [5]. Moreover exposure to environmental tobacco smoke (ETS) is still a concerning problem. Smoking is a cause of a number of serious (and often preventable) diseases like cancers of the lungs, pancreas, esophagus, bladder and oral cavity, cardiovascular, respiratory system and gastrointestinal diseases, impaired fertility and overall acceleration of the aging process [6]. This is because tobacco smoke (both its gas phase and its particulate phase)—in addition to the main and harmless components, which are nitrogen, oxygen and carbon dioxide (and account for 85% of the weight of tobacco smoke) [7]—is a toxic, mutagenic and carcinogenic mixture of more than 5000 chemicals, of which over 30 are known human carcinogens [8]. These are, among others: Tobacco-specific nitrosamines (nitrosoanabasine (NAB), nitrosoanatabine (NAT), 4-(methylnitrosamino)-1-(3-pyridyl)-1-butanone (NNK) and nitrosonornicotine (NNN). Other nitrosamines include 4-(methylnitrosamino)-1-(3-pyridyl)-1-butanol (NNAL), 4-(methylnitrosamino)-4-(3-pyridyl)-1-butanol (isoNNAL) and 4-(methylnitrosamino)-4-3-pyridyl) butyric acid (iso-NNAC) [9]), phenol, cresol, β-naphthylamine, N-nitrosonornicotine, benzo-a-pyrene, nickel, arsenic, polonium 210, indole and carbazole, tar and polycyclic aromatic hydrocarbons [7]. These substances are the major causes of human cancer [10]. In addition, tobacco smoke contains a number of substances that are irritating and toxic to the ciliary epithelium of the respiratory system: Hydrogen cyanide, acetaldehyde, acrolein, ammonia, formaldehyde, nitrogen oxides, hydrazine and vinyl chloride [7]. Other forms of nicotine delivery, such as electronic cigarettes (ECs), which are alternative smokeless devices, are also not free from those substances. However, many studies show that they are still much safer in this regard, because the content of hazardous compounds is 9—450 times lower in EC vapour than in conventional cigarettes [11]. Of course, this does not mean that they do not affect the health of the smoker. It should not be forgotten that e-cigarette liquids also contain nicotine—this substance is highly addictive and, furthermore, can harm brain development (which continues until about age 25 years) [12].

The main aim of this study was to review recent discoveries about environmental tobacco exposure in the context of the nervous system from January 2015 to December 2020. The secondary aims of this study were to determine what impact maternal smoking during pregnancy has on the nervous system, how tobacco influences cognition and brain functions, what are new discoveries in the field of its relation to neurovascular diseases, what are the outcomes of mixing tobacco with other stimulants, what are radiological findings in tobacco users and whether there is any relation between grand-maternal smoking and offspring health.

## 2. Materials and Methods

### 2.1. Search Strategy

**Figure 1:**
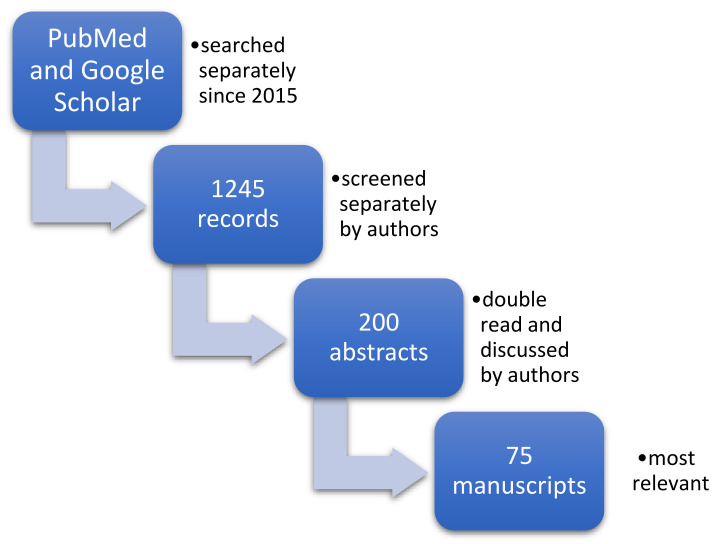
The process of data collection.

To prepare this manuscript, the search engine PubMed and google scholar were used from the beginning of 2015 (Figure 1). With the uses of keywords as fallows: Tobacco, cigarette, nervous system, brain, morphology, function, grandmother, smoking, pregnancy, father, development, paternal, prenatal. In addition to using singular key words, to find the most relevant records the authors used PubMed Advanced Search Builder as well. Used advanced queries were as follows: (((tobacco) AND (smoke)) AND (nervous)) AND (morphology); ((tobacco) OR (cigarette)) AND (((nervous system) OR (brain)) AND ((morphology) OR (function))). To find most relevant paper, two analysts (WH and AŻ) were working separately by screening the search engines.

### 2.2. Data Extraction

As a result, 1245 records were identified and screened separately by authors. Each of the analysts prepared their own list of records identified as relevant to the study. Then these record’s lists were double read by both of them and 200 abstracts were found to be relevant to the subject. Then full text manuscripts were acquired. Authors prepared a small database of full-text manuscripts and indexed them in the index table to organize work so that each article got their own ID (Primary Key) used for authors’ personal communication. Then all articles were read independently by both analysts. At this stage each abstract was, for practical purposes, initially assigned to one of the categories that were used to accelerate further work (like: Introduction, animal-based, human-based, etc.).

### 2.3. Qualitive Analysis and Synthesis

Each analyst worked independently and prepared their own list of relevant full-text manuscript. Both lists were compered and by discussion 75 publications were found to be the most relevant to the study and included in this review. Manuscripts were assessed for both the applicability to the aim of the review and recency of publication.

## 3. Results

In this review, the authors present results covering seven different areas of tobacco impact on human health and for four of them animal-based studies are discussed as well. The covered areas are nervous system development, neurotransmitters, cognition and brain functions, neurovascular diseases, stimulants, neuroradiology and grand-maternal and paternal smoking. The results describe tobacco influence from the earliest moments of life up to its impact to neurodegenerative diseases commonly encountered in late stages of it. The authors also present discoveries on various levels of organization from cytological through functional level to emotional and mental. The most important findings are listed in Table 1.

### 3.1. Nervous System Development, Embryology and Histology (Tobacco Impact on Formation and Differentiation of Neural Basic Structures)

Tobacco product use is a major public health problem all over the world—smoking is a recognized risk factor for many diseases, including morphological changes in a nervous system. There are currently about 1.1 billion daily smokers worldwide (according to WHO) [13]. This exposes more than one third of the adult population to passive smoking and causes about six million deaths every year (including 600,000 deaths from secondhand smoke). Tobacco smoke contains over 7000 chemicals, including more than 4500 toxins and about 70 carcinogens. Researchers suggest that tobacco toxins (including vinyl chloride—a risk factor for brain cancer, hydrogen cyanide, and arsenic) influence brain volume. Approximately 15% of cigarette smoke is absorbed by the smoker. 85% is released into the atmosphere. Cigarette smoke is more harmful to intensively developing organisms—babies, infants and developing fetuses—and that is why a prenatal exposure to tobacco smoke is particularly burdened with consequences: Increased risk of spontaneous abortion, low birth weight, sudden infant death syndrome (SIDS), neurodevelopmental, mental and cardiovascular diseases in children. Immature lungs and immune system increase the risk of developing acute and chronic diseases in the future. The studies conducted so far show that exposure to maternal smoking impairs the structural development of the fetus brain [14]. Morphological changes acquired in the prenatal stage cause cognitive decline, increased risk of dementia, difficulties with emotional regulation and impaired working memory in postnatal life.

Similarly, maternal smoking during pregnancy was proved to affect the descendant’s gene expression. In one of the observational studies, in which 39 pregnant women were included after assessing their cotinine, the nicotine metabolite and the genes *RELN*, *PLP1*, *MBP* and *BDNF*, down-regulation was observed in samples collected from umbilical blood and, since those genes control brain growth, it might be stated that tobacco smoke can by one of the causes of impaired neurological development and that it impacts fetal brain programming [15]. Similarly in a very large study (9000 adults) perinatal smoke exposure was associated with a negative long-term effect on sensory cortices [14].

#### Animal-Based Studies

Not only does prenatal exposure to tobacco lead to some impairments in neurodevelopment but research carried on out on animals in which rats were exposed to tobacco in early postnatal period showed that about 800 proteins (37.5%) of the prefrontal cortex were significantly responsive to environmental tobacco smoke, 716 of which, mainly enriched in association with neurons and synapses, presented an increase in abundance. In contrast, the remaining 84 of proteins, largely mitochondrial associated, presented a decrease. Consequently, by using ion-mobility-enhanced data-independent mass spectrometry, the study revealed prefrontal cortex vulnerability to ETS during early neurodevelopment [16].

### 3.2. Neurotransmitters and Tobacco—Receptors, Neurotransmitters, Signalling Pathways

Acetylcholine receptors, which are in many cells of the human body, can be divided into nicotinic (nAChR) and muscarinic (mAChR). While the nicotinic one has an affinity to both the nicotine and the acetylcholine, a degree of it remains dependent on a receptor’s structure and varies from very high affinity to both substances in an α4β2 subtype, which is widely encountered in the central nervous system, to low nicotine affinity levels in receptors at neuromuscular junctions. In addition, Nicotine cannot be quickly eliminated from the synaptic cleft which, due to prolonged triggering of receptors, firstly stimulates and then leads to desensitisation of post synaptic neurons and consequently results in difficulty of transmitting signals [4,17]. Moreover, this desensitisation can be allosterically aggravated and prolonged due to the presence of menthol, widely used as a flavour additive in cigarettes [18]. It should be also considered that nACh receptors are present in cortical and limbic structures which relate to the addiction process (particularly cerebral cortex, amygdala, nucleus accumbens, hippocampus, ventral tegmental area (VTA)). In normal state, VTA neurons are controlled by cholinergic neurons from the pedunculopontine nucleus (PPN) and the lateral dorsal tegmental nucleus (LDTN). However, in the case of nicotine intoxication, the substance stimulates the nACh receptors and indirectly modulates LDTN cholinergic neurons. Therefore, nicotine by stimulating nACh receptors of the VTA’s dopaminergic neurons causes prolonged bursts that then release dopamine to the nucleus accumbens which, as a part of the reward system, is said to participate in translating motivation to action and consequently may be an important part for the disproportionate motivational drive related to nicotine and other drug abuse [5,19]. As well as that, a meta-analysis of molecular imaging studies on tobacco smoking and dopaminergic functions of brain revealed a lowered transporter for dopamine but unaffected D2 dopamine receptor availability in smokers. The authors suspect that these findings may either by for pathophysiological reasons, for some people are more susceptible to tobacco dependence, or a result of long-lasting exposition to nicotine [20].

#### Animal-Based Studies

In addition, an experiment on HIV-1 transgenic rat was conducted to study drug addiction in patients who were on active anti-retroviral therapy. Received data indicate that HIV-1 proteins might be responsible for alteration of subunits concerning the nACh receptors’ expression and may commit to susceptibility to nicotine and smoking [21]. Similarly, a research carried on four groups of rats (12–17 dams each) was conducted to revise the risk of neurodevelopmental disorders caused by maternal smoking during pregnancy. The study assessed the impact on brain regions including main Ach and 5HT projections with corresponding cell bodies. Therefore, to measure this activity, choline acetyltransferase (ChAT), concentration of presynaptic high-affinity choline transporters (HC3) and a4b2 nAChRs were evaluated. The study appears to be important because it assessed not only the nicotine itself but also the entire tobacco smoke extract (TSE), which makes correspond more closely to real-life situations. The authors revealed that the TSE adverse effects on neural development exceeded those typically attributed to nicotine itself present in mixture. Presynaptic activity (reduced HC3/ChAT ratio) was impaired more strongly because of TSE than nicotine alone. Moreover, greater difference was exposed for the impact to nACh receptors, opposed to the increase during exposition of only nicotine. In groups that were subjected to TSE, the receptor decreased, which was associated with an exacerbation of functional consequences of presynaptic deficit. Overall, the study indicated that “the complex mixture of compounds in tobacco smoke contributes significantly to developmental neurotoxicity over and above the effects of nicotine” [22]. These discoveries lead professor Slotkin to a second experiment to estimate the critical period in which TSE has the greatest impact on neural development. By administrating TSE in three distinct 10-day moments (premating, early gestation, late gestation) it was found that there was no “critical period” for effects of low-level tobacco smoke. While the sensitivity to TSE was somehow dependent to the developmental stage, the findings strongly supported the need to avoid smoke exposure both during pregnancy and prior to it [23].

### 3.3. Impact on Cognition, Higher-Order Brain Functions—Perception, Attention, Memory, (Nicotine Receptors in Hippocampus and Limbic Structure)

#### 3.3.1. Impact on Cognition and Memory

Owing to the nACh receptor relationship with cognition there is an undoubted connection between nicotine and higher-order brain functionality. Tobacco’s influence on cognition can be divided into acute and chronic. At first, in acute smoking the nicotine is capable of improvement of hippocampus-dependent learning as well as memory and attention. However, secondly, in chronic smoking hippocampus-dependent learning is depressed once the nicotine tolerance is developed and even can be a risk factor for Alzheimer’s disease [3]. Moreover, one study on 628 inhabitants of the Haidian district of Beijing found that smokers had significantly lower immediate and delayed memory as well as whole cognitive scores on the RBANS total score, which is a repeatable battery for the assessment of the neuropsychological status composed of 12 subtests [24]. In addition, one study, carried out on a cohort of 720 mother–child pairs, assessed exposure to second-hand smoke (SHS) during pregnancy and then children neurodevelopment at 24 months old. To analyse the impact of tobacco, the mental developmental index (MDI) and psychomotor developmental index were measured and compared with urine cotinine level, which is a predominant metabolite of nicotine. The study excluded actively smoking mothers, focusing on environmental exposition. The study showed that cognitive development of infants decreased significantly with increasing maternal cotinine level [25]. Further research in this field aims to evaluate the influence of tobacco on brain functions. A cohort study carried out on 1739 mother–newborn pairs was conducted and aimed at measuring how prenatal exposure to alcohol and tobacco affects offspring neurological activity. Therefore, EEG examination was performed. Consequently, the infant whose parent were continuously exposed to higher doses of tobacco during gestation had significantly decreased right–central and right-parietal beta, and low gamma and gamma EEG power compared to groups with lower smoke doses. The authors claim that the outcomes of their study may suggest the abnormal maturation of cortical networks. Overall, the findings showed that any level of prenatal tobacco exposure has an association with new-born brain activity [26]. Additionally to this, a study measuring prenatal tobacco exposure on 239 children aged 8 uncovered an association between pregnancy-time cotinine, a nicotine metabolite, level and executive functions evaluated via the Behaviour Rating Inventory of Executive Function. The rating’s area associated with tobacco included initiation (ability to independently begin tasks), working memory (ability to retain critical information to complete a task) and organization (ability to organize work or storage spaces). Furthermore, the findings of this study supported that even exposure to low-dose second-hand tobacco during pregnancy may affect children’s development [27]. Nonetheless it should be also mentioned that one of the studies found no statistically significant effects of smoking on cognition [28]. In one review, several studies on nicotine impact concerning tobacco were discussed; in one of its subsections the authors discuss the difference of cognition after administration of nicotine between previously non-smokers and abstinent smokers. The authors conclude with the idea that nicotine has differential effects on cognition depending on smoking history and its withdrawal status [29]. Single studies of evoked potentials in smokers suggest bioelectric dysfunction at the cortical and subcortical levels. An analysis of motor-evoked potentials (MEP) using transcranial magnetic stimulation showed lower motor cortex activation (lower MEP amplitude) in tobacco smokers compared with control group [30]. Jawiński et al. analysed cortical auditory evoked potentials in smokers and revealed higher N1-P2 amplitudes in never-smokers compared to ex- and current smokers. Moreover, they observed pack years and number of cigarettes consumed per day to be inversely correlated with evoked potential amplitudes in current smokers [31]. Studies on the N200 component of event-related potentials (EPR) have shown a reduced amplitude in smokers compared to non-smokers. It seems that the N200 wave is an initial component of ERP and, in a way, is “ready” to perform a cognitive task. Its amplitude reduction suggests a subclinical disturbance of cognitive control and, consequently, may lead to cognitive impairment [32].

##### Animal-Based Studies

One of the studies on adult mice exposed to a very low dose of tobacco smoke over a long period of time found the reduction of immature neurons in the hippocampal dentate gyrus and, as well as nicotine, the contribution of inflammatory mediators was suggested [33]. In a similar way, a study conducted on 24 rats shows that exposure to waterpipe tobacco smoke for 1 h twice daily during lactation leads to impaired long-term memory, associated with reduced level of brain-derived neurotrophic factor in hippocampus in the offspring of dam. In particular, the results of a memory test conducted after 24 h were impaired, but without significant impact on the short-term one. Authors suggest that this may be due to different pathways (molecular and synaptic) and moreover to the fact that hypothalamus is developed during the first two weeks of postnatal life in these animals [34]. In addition, a second study on approximately 28 rats exposed to waterpipe smoke for 60 min a day five days a week for four weeks by using the whole-body exposure apparatus was conducted. To evaluate spatial learning and memory the radial arm water maze test was performed (RAWM). Furthermore after four weeks of experiment hippocampus dissection was caried out and levels of oxidative stress enzymes were quantified. Waterpipe tobacco smoking (WTS) was not associated with impairment of learning but, on the contrary, it negatively affected short and long-term memory. Further research found that WTS caused reduction in activity of antioxidant enzymes [35]. These findings might be juxtaposed with the other research where Astaxanthin administration, which is a carotenoid present in many kinds of marine organisms, was found to reverse ETS-induced cognitive deficits in mice due to normalisation of oxidative stress markers and ceasing of inflammation. Furthermore, astaxanthin administration lead to an attenuation of p38 MAPK stress-activated kinase, which is associated with a neuronal apoptosis [36].

#### 3.3.2. Neurodegenerative Diseases

##### Animal-Based Studies

Neurodegenerative diseases are a group of congenital or acquired progressive diseases of the nervous system. Their common pathological mechanism is the progressive reduction in the number of nervous cells. Consequently, before the first symptoms are developed, because of the loss of a significant number of neurons (or of damage to a specific part of the central nervous system), the process leading to their appearance begins much earlier and is asymptomatic for a long time. Ultimately, several symptoms of the neurological damage that occur affect motor and cognitive functions [37]. A study carried out on mice showed reductions in myelin staining intensity and narrowing of the corpus callosum caused by the inhibitory effect of tobacco smoke on the expression of genes needed for myelin synthesis and maintenance [38]. A particularly high impact on myelination disorders involves an exposure to environmental tobacco smoke in the early postnatal period (especially the first two weeks) [39].

##### Human-Based Studies

Tobacco use has been identified by accumulating evidence as an important modifiable risk factor for developing cognitive decline such as Alzheimer’s disease but the exact mechanisms of it remains unclear [40]. In particular, tobacco was associated with faster decline in a functional performance in mildly cognitive impaired participants, which is thought to be an Alzheimer’s disease prodromal state, along with steeper decline in entorhinal cortex volume. Similarly in other research, tobacco was correlated with memory impairment, hippocamp deterioration and brain glucose metabolism decrease. However, neither of those two found an association between CSF AD pathologies (Aβ42, t-tau and p-tau) and history of smoking [40,41]. In addition, due to tobacco’s effect on the loss of white and grey matter, the relative brain age of the smokers is older than non-smokers. It is thought that certain behavioural conditions are associated with accelerated atrophy in certain brain regions. Smokers have significantly smaller grey matter volume, lowered grey matter density and a greater rate of atrophy in regions that show morphological abnormalities in the early stages of Alzheimer’s disease (compared to non-smokers). It is still unclear how smoking and alcohol consumption is associated with brain structural aging, especially when the morphology of all the brain regions is considered [42]. Likewise, meta-analysis studies showed that smoking damages white matter structural integrity, which is a risk factor for Alzheimer’s disease. Apart from AD, tobacco was also found to be a risk factor for multiple sclerosis [43], along with reducing time to conversion to secondary progressive MS when smoking after diagnosis [44].

#### 3.3.3. Protective Influence of Nicotine on Dopaminergic System in Parkinson’s Disease

Parkinson’s disease is the fastest growing neurodegenerative disease. The number of patients grew worldwide from 2.5 million in 1990 to 6.1 million in 2016 [45]. The aging of the population is the main reason for this significant increase, but among other things, it could be due to a decline in the popularity of smoking addiction. Despite the many adverse effects of smoking, there are many studies that show an inverse relationship between tobacco use and the development and death of Parkinson’s disease. Though there is compelling statistical evidence, the underlying biological basis for this relationship is still unknown. The first suggestion of a protective effect of smoking against the development of Parkinson’s disease was made by Dorn 60 years ago [46,47]. The studies were carried out on different groups and at different observation times, all leading to one conclusion that smoking (in various forms—cigarettes, pipes, cigars) reduces the risk of developing and dying from Parkinson’s disease, while current smokers have a lower risk than former. One such study was a longitudinal cohort study involving 34,439 males followed between 1951 and 2016. It was conducted by Mappin-Kasirer et al. This study reported a total of 25,379 deaths, including 283 with Parkinson’s disease as the leading cause of death. The death rates from Parkinson’s disease among current smokers at the beginning were 34.7% lower than those among non-smokers. Compared with non-smokers, current smokers at baseline or during follow-up had a 30% and 40% lower risk of Parkinson’s disease, respectively. Overall, this study found that current tobacco smoking, smoking amount and time elapsed since cessation were all associated with the risk of Parkinson’s disease [46].

#### 3.3.4. Insomnia and Affective Disorders

Both stimulants and sleep disorders have a negative impact on health. Low sleep quality has been associated with substance use (including smoking) and reversed causation from insomnia to substance use (individuals with poor sleep quality have a stronger inclination for later substance use [48]), but the direct link remains unclear [49]. Interestingly, for smoking, studies have shown a moderate genetic correlation with insomnia [50].

Nicotinism causes a few sleep disorders. Compared to individuals who do not smoke cigarettes, smokers are more likely to experience sleep apnoea, sleep disordered breathing, insomnia and poor sleep quality (increased difficulty maintaining sleep, increased sleep latency, daytime sleepiness, shorter sleep duration), which are risk factors for many chronic diseases like obesity, cardiovascular diseases, diabetes and depression. These conditions are further exacerbated by tobacco use. Therefore, it is kind of a vicious circle—smoking can cause sleep disturbances, which in turn exacerbate other diseases caused by smoking which are serious non-communicable health problems worldwide [51].

### 3.4. Impact of Tobacco on Other Neurovascular Diseases (Stroke, Aneurysm, Atherosclerosis), Migraine + Nicotinism—Significant Increase in Risk of Severe Strokes

Smoking, both active and passive is a well-known health hazard and risk factor of all cardiovascular diseases. The risk of cardiovascular diseases depends on the number of cigarettes smoked, but it is not a linear relationship. Studies show that even smoking only one cigarette daily increases the risk of developing coronary artery disease and stroke [52]. The pathogenesis of this relationship is multifactorial, but recent clinical and experimental findings support the assumption that tobacco smoke exposure increases oxidative stress as a potential mechanism for initiating cardiovascular dysfunction: Cigarette smoke (both active and environmental) causes changes in the endothelium of blood vessels, increases oxidative stress, reduces nitric oxide (NO) production, increases insulin resistance and activates the sympathetic system, which affects the development of arterial hypertension and atherosclerosis, as well as atrial fibrillation and metabolic disorders such as obesity [53]. It should also be mentioned that in the short term, nicotine increases energy spending and reduces appetite and some research has revealed that smokers may have lower body weight than non-smokers; some other evidence states that people who smoke heavily tend to have heavier body weight than people who smoke less. This is explained by the association of smoking with other risky behaviors such as lesser physical activity or worse diet. Smoking in the long term may contribute to insulin resistance and increased risk of metabolic syndrome. In young adults increased probability for weight loss was described among occasional smokers [29,54]. As a consequence, cardiovascular diseases develop, e.g., stroke, acute coronary syndrome, coronary artery disease. The development of atherosclerosis affects all arterial vessels—coronary arteries, cerebral arteries, renal arteries, peripheral arteries and the aorta. Therefore, smoking is one of the most serious (but preventable at the same time) factors in the development of non-communicable diseases [55].

Exposure to cigarette smoke increases the hazard of rupture of atherosclerotic plaque, thereby promoting thrombus formation and leading to an acute coronary syndrome (including sudden cardiac death). Furthermore, smoking promotes coronary artery spasm. Moreover, studies show tobacco use is a relevant risk factor for stroke, cerebral aneurysms, subarachnoid haemorrhage (relative risk, 2.93; 95% confidence interval, 2.48–3.46) and cerebral infarction (relative risk, 1.92; 95% confidence interval, 1.71–2.16) in both sexes [52].

Smoking is a potent risk factor for abdominal aortic aneurysm. A meta-analysis showed a fivefold increase in the risk of an abdominal aortic aneurysm for current smokers and a doubling of the risk for former smokers as against individuals who have never smoked [52].

Avoidance of headache triggers plays an important aspect in the treatment of migraines. Meanwhile, tobacco awareness and avoidance are rarely emphasized as part of that treatment schedule. Taylor RF conducted a study that was aimed at examining the various types of tobacco products to which migraineurs are exposed and studying the mechanisms by which nicotine exposure causes headaches [56]. As the result, he states, that there are some conflicting data that support the validity of patient history of exposure to tobacco smoke as migraine headaches trigger but prospective controlled studies are needed. The exact pathomechanism is not yet known [56].

### 3.5. Nicotine and Other Drugs and Stimulants—Additive Effect

Combining stimulants is a very common phenomenon; therefore, the analysis and consideration of their coincidental impact are extremely important. Globally, cannabis and tobacco (not to mention combining tobacco with alcohol) are commonly mixed together and smoked jointly (a practice called “mulling”). There is still limited knowledge about impact of co-use on metabolic changes and neural tissue development, but this has been associated already with adverse effects on educational outcomes, impaired emotional and physical functioning, lower cognitive performance and increased risk of substance-use disorders and addiction [57]. Moreover, studies show that combining stimulants has a particular effect on reducing GMV: There is a negative relation between the number of substances used (e.g., alcohol, cocaine, tobacco, cannabis) and the volume of the specific brain area—dorsal medial prefrontal cortex—and those alterations are related to the number of substances used, while ventrolateral prefrontal cortex pathology is specifically associated with tobacco use [58,59]. Tobacco is sometimes mixed not only with alcohol but also with narcotics; in particular the effect of prenatal combining tobacco with marijuana was studied. The study indicated an indirect association between this combination and autonomic functioning during the second year of life [60]. Similarly, a connection between prenatal cannabis exposure (mostly combined with tobacco) and cortical thickness was suggested [61]. Nevertheless, consequences of recurrent nicotine and cannabis co-stimulation during adolescence still remain understudied [62].

#### Animal-Based Studies

Mixing tobacco and alcohol can multiply negative outcomes for the nervous system and such outcomes are supported by several studies. In the first animal-based study, different group of rats were exposed to alcohol, tobacco and both. The level of the hippocampus’ intracellular reactive oxygen species along with inflammatory parameters was significantly increased in the mixed group, but decreased the brain-derived neurotrophic factor, which plays a role in synaptic plasticity and neuronal survival [63]. Similar outcomes were also present in the outcomes of the second study, which found that combining alcohol and tobacco increased rats’ locomotive activity, and that combining nicotine and alcohol has an additional anxiolytic effect and leads to the growth of grooming behaviours [64].

### 3.6. Neuroradiological Examination

Evidence from the studies carried out so far using voxel-based morphometry (VBM) revealed that widespread brain regions are involved in chronic smoking and localization of gray matter abnormalities caused by tobacco smoke is heterogeneous. There are many researches linking tobacco use to changes in specific areas of the brain. Vňuková M et al. analyze studies which used VBM to show differences in gray matter volume (GMV) between smokers and non-smokers—smoking decreases GMV in most brain areas, but also some sex-specific dependencies were discovered: The thalamus and cerebellum were affected in both sexes, but reduction of GMV in the olfactory gyrus was found only in male smokers [65]. Similarly, in one study, GMV of bilateral anterior insula were negatively correlated with nicotine dependance [66]. Zhong J et al. carried out a meta-analysis comprising eleven studies with 686 chronic cigarette smokers and 1024 nonsmokers [67]. The findings were converging and reveal a characteristic neuroanatomical pattern in chronic smokers: The chronic smokers showed a considerable gray matter reduction in the bilateral prefrontal cortex and gray matter increase in the right lingual cortex. Furthermore, a correlation between the number of smoking years, cigarettes per day and abnormalities in gray matter structure was demonstrated. Salminen LE et al. analyzed available magnetic resonance imaging data from the UK Biobank (UKB), which is currently the largest prospective study of aging and collecting information about health, physical state and lifestyle for over 500,000 middle-aged people and other adults living in the United Kingdom. They examined white and gray matter volume and structure in subgroups of perinatal-exposed and unexposed to secondhand smoke individuals. The study revealed significant, system-specific effects of perinatal exposure to secondhand smoke on sensory brain structures later in life [14]. Similarly, in a magnetic resonance imaging study, nicotine potential of destruction to white matter and neurons was confirmed [68]. Moreover, nicotine deprivation in smokers was associated, in an MRI study, with lower brain activity in its areas responsible for attention contrary to areas involved with craving, which showed lower activity when nicotine was present [69].

### 3.7. Grandmothers and Paternal Tobocco Smoke and Its Impact on Childen’s Health

It is a truth universally acknowledged that smoking during pregnancy has a detrimental effect on offspring development and health. However, some interesting findings have been revealed by new studies concerning not only maternal smoking but also grand-maternal and paternal. One of the studies found a strong negative association between the paternal grandmother’s smoking during her pregnancy and her grandson’s chance of developing early onset myopia [70]. What is more, grandmothers smoking, while mothers of the offspring were in the womb, was associated with asthma and nasal allergies in their grandchildren and, what is even more interesting, fathers’ smoking before the 15 years age (in puberty) and maternal smoking during pregnancy was associated with higher risk of asthma without allergies in their offspring. The authors speculate that, while parental smoking has a detrimental effect on lung development, grandmothers smoking might be associated with epigenetics and dysregulation of immune development [71]. Similarly, another cohort study on a large-scale population associated grandmothers smoking with grandchildren asthma [72]. In addition similar results were found in a study covering a cohort of 1629 newborns that revealed association between paternal tobacco smoke and child’s asthma development at six years old; moreover, it was neither associated with total IgE levels nor with allergen sensitization. The authors suggest an IgE-independent mechanism [73].

## 4. Discussion

In recent years, much effort has been put into describing, revealing and uncovering new detrimental aspects of tobacco to human life. Both animal and human-based studies have contributed equally to progress in this area of knowledge. In animal-based studies, particular attention has been paid to molecular and prenatal aspects of studies and the most important conclusion that can be derived from them is that tobacco appears to be more harmful than nicotine itself, whereas human-based studies focused on higher-order brain functions, concluding that tobacco can significantly contribute to many neurodegenerative diseases with the interesting exception of Parkinson’s diseases. Presented studies are not nearly the tip of the iceberg in those fields. It seems that not only the morphology but even the function of the brain is impaired by tobacco. According to NS development, particularly interesting discoveries covered the mother’s smoking and child’s memory impairment [14] and (from animal studies) early post-natal exposition to tobacco [16]. These two fields relate to each other due to the fact that one can be a consequence of the other. Typically, if a mother does not cease smoking during pregnancy, it would be rather unlikely for her to cease later and this could possibly lead to an accumulation of detrimental effects. However, further studies are required in these fields, especially transcription from animal studies to human-based ones. Subsequently, in the neurotransmitters subsection, we presented cytological and molecular level findings in the area of tobacco influence. In this field, particular attention should be paid to the dualistic nicotine [4,17] impact on synaptic cleft the first triggering signal transmission but in a longer period of time there is an impairment of transmission due to desensitization that is an important basis for further research and helps to understand tobacco’s impact on neurodegenerative diseases described in the following paragraphs. Moreover some animal-based studies described in this subsection bring interesting results about more harmful effect of whole tobacco compared to nicotine itself [22]. According to cognition and higher-order subsection, it is important to distinguish both the acute and chronic tobacco influence. While in the acute sort, nicotine is capable of memory improvement, in longer periods of time it can contribute to Alzheimer’s diseases and impair memory [3,24,25,40]. Some animal studies support the protective role of Astaxanthin to tobacco smoke and its impact on normalization of oxidative stress markers. This could be an interesting way of reversing detrimental effect of ETS. However, further research is needed, especially human-based research [35,36]. Particularly interesting results were found concerning tobacco’s relation to Parkinson’s disease and an inverse relationship between tobacco use and development of diseases [45,46,47]. In addition, some absorbing research studied nicotine’s contribution to sleep disorders, especially sleep disorder breathing, insomnia and poor sleep quality [51]. One of the most interesting findings was in the area of grand-maternal smoking. The presented results described an implication between grandmother’s smoking and grandchildren’ asthma and allergies so that smoking contributes to diseases beyond one generation [71,72,73].

### Strengths and Limitations

The authors made every effort to find and screen as many records as possible and attempted to discuss the subject at length. During the preparation of this manuscript, the authors tried to adopt and follow in a simplified way some of the systemic review’s principles [74]; in particular:

- The manuscript was prepared by a team not a single person.
- Record screening, evaluation of eligibility and data extraction were done by two people working independently at each stage and then synthesized.
- The review was based on independent assessments and the authors declared no conflict of interest.

However, it has to be stated clearly that this study is not a systemic review and has never aimed to be one. Due to a lack of resources, the authors could not cover every single aspect of tobacco’s influence on the nervous system and focused only on the main areas predefined in the process of the manuscript’s development. It also has to be mentioned that the article presents some of the animal-based studies and this type of study cannot by directly compared to human beings without further research.

## 5. Conclusions

To summarize, we would like to emphasize that current studies appear to show that tobacco can be more harmful than pure nicotine itself, and exposure to it, particularly in early stages of development, is certainly undesirable. Tobacco contributes particularly to neurodegenerative diseases, impacts cognition and relates to the grey matter volume of the brain. For future perspectives, it appears to be particularly of interest to better control the effects of tobacco and consider smoking as a major public health problem. The authors believe that particular emphasis should be put on childbirth schools and early school education so the future parents will be aware of tobacco smoke exposure’s varied detrimental effects on many aspects of fetal development as early as possible.

## Figures and Tables

**Table 1:** Most important findings of this review.

Subsection	Human Based	Animal Based
3.1 NS development	Perinatal: Maternal smoking impairs brain development and sensory cortices	Postnatal: 800 proteins of prefrontal cortex responses to tobacco
3.2 Neurotransmitters	Lowered transporter for dopamine and unaffected D2 receptor in smokers	HIV-1 proteins may commit to susceptibility for nicotine and smoking; tobacco has more adverse effect than pure nicotine
3.3 cognition and brain functions	Perinatal: Smoking impairs infant postnatal development; Postnatal: Chronic smoking impairs memory; impact to cognition depends on prior smoking history; ETS contributes to Alzheimer disease and multiple sclerosis and sleep disorders; Tobacco alleviate Parkinson’s disease symptoms	Postnatal: Smoking during lactations impairs offspring memory; ETS impairs myelination of NS; Tobacco causes reduction in antioxidant enzymes
3.4 Neurovascular diseases	Risk depends on number of cigarettes smoked but its not linear; tobacco increases oxidative stress	-
3.5 Tobacco and stimulants	Mixing impairs emotional and physical functioning, reduce grey matter volume (GMV);	Mixing contributes to oxidative stress in hippocampus and decreases brain-derived neurotrophic factor
3.6 Neuroradiology	Postnatal: Smoking reduces GMV; nicotine contributes to destruction of white matter and neurons; nicotine deprivation is associates with lower activity in attention brain areas; Perinatal: effect on sensory brain structures	-
3.7 Paternal and grand-maternal smoking	Perinatal: Grandmothers and paternal smoking associated with offspring’s asthma	-

## Data Availability

Not applicable.
